# Stereotactic laser ablation in neuro-oncology - A survey among European neurosurgeons

**DOI:** 10.1016/j.bas.2023.101749

**Published:** 2023-04-28

**Authors:** Ilaria Viozzi, Maroeska M. Rovers, Christiaan G. Overduin, Mark ter Laan

**Affiliations:** a Department of Neurosurgery, Radboud University Medical Center, Radboud Institute for Health Sciences, 6525 GA, Nijmegen, The Netherlands; b Department of Medical Imaging, Radboud University Medical Center, Radboud Institute for Health Sciences, 6525 GA, Nijmegen, The Netherlands

**Keywords:** Stereotactic laser ablation, Neuro-oncology, Glioblastoma, Survey

## Abstract

**Introduction:**

In the last decades, the application of stereotactic laser ablation (SLA) for the treatment of intracranial tumours has been growing, even though comparative trials are lacking. Our aim was to investigate the familiarity with SLA of neurosurgeons in Europe and their opinion regarding potential neuro-oncological indications. Furthermore, we investigated treatment preferences and variability for three exemplar neuro-oncological cases and willingness to refer for SLA.

**Material and methods:**

A 26-questions survey was mailed to members of the EANS neuro-oncology section. We presented three clinical cases of respectively deep-seated glioblastoma, recurrent metastasis and recurrent glioblastoma. Descriptive statistics was applied to report results.

**Results:**

110 respondents completed all questions. Recurrent glioblastoma and recurrent metastases were regarded as the most feasible indications for SLA (chosen by 69% and 58% of the respondents) followed by newly diagnosed high-grade gliomas (31%). Seventy percent of respondents would refer patients for SLA. The majority of respondents would consider SLA as a treatment option for all three presented cases: 79% for the deep-seated glioblastoma case, 65% for the recurrent metastasis case and 76% for the recurrent glioblastoma case. Among respondents who wouldn't consider SLA, preference for standard treatment and lack of clinical evidence were reported as the main reasons.

**Conclusions:**

Most of respondents considered SLA as a treatment option for recurrent glioblastoma, recurrent metastases and newly diagnosed deep-seated glioblastoma. At the moment the current evidence to support such a treatment is very low. Comparative prospective trials are needed to support the use of SLA and determine proper indications.

## Highlights

• Responders of this survey showed high willingness to refer patients for stereotactic laser ablation (SLA).
• Recurrent high-grade glioma's and recurrent metastases stand out as the most suitable indications for SLA.
• Insufficient clinical evidence remains a big concern reported regarding SLA.

## 1 Introduction

In the last decades, the application of stereotactic laser ablation (SLA) for treatment of intracranial tumours has been growing (Ashraf et al., 2018). In SLA, laser light is transmitted from the generator to the tumour through the use of optical fibres causing local thermal damage. (Franck et al., 2016). Through a short skin incision and a small burr-hole, a fiber optic laser probe is stereotactically placed in the target tissue. When activated, laser light causes thermal damage in the target tissue leading to coagulative necrosisand ultimately to cell death. Since the first reports, many advancements have been made, the most important being the integration of SLA with magnetic resonance imaging thermography (tMRI), which allows to accurately estimate thermal damage in real time and ablate the target tissue while minimizing thermal damage to adjacent structures (Munier et al., 2018). Even if not a first-line option in treating intracranial pathology, SLA has been used for cases in which tumours are difficult to access surgically, in patients with a greater surgical risk or in case of recurrence and repeat resections (Ivan et al., 2016).

So far, studies on SLA mainly consist of retrospective or prospective small case-series and there is no high-quality evidence comparing SLA with standard of care. While in the United States SLA is already applied in many institutions, CE (Conformité Européenne) marking is only available since March 2018, and the treatment is offered only in a few centres in Europe. Despite the lack of evidence, the application of SLA in neuro oncology is growing, as shown from the increasing number of publications on the topic over the years (Ashraf et al., 2018).

The aim of this study was to investigate European neurosurgeons’ familiarity with SLA and their opinions concerning possible neuro-oncological indications for this treatment. Furthermore, we wanted to explore treatment variability for exemplar cases and willingness to refer patients for SLA.

## 2 Material and methods

**Fig. 1:**
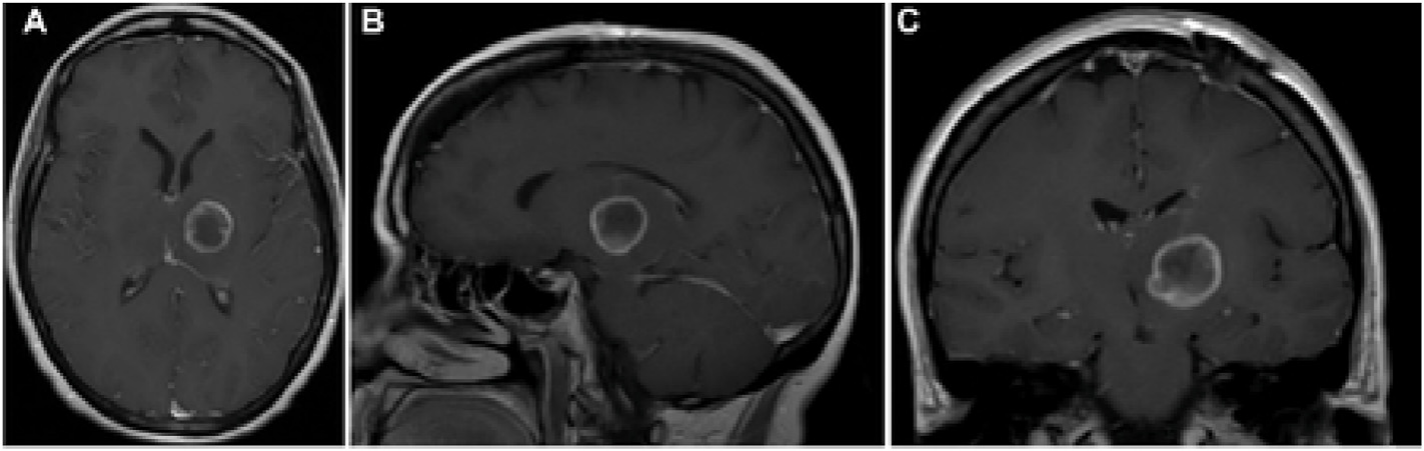
nGBM case - A 40 years old male presents with a lesion in the thalamus. A stereotactic biopsy has been performed and histopathological analysis shows a IDH wild type glioblastoma (WHO gr 4). No targetable mutations were found. Patient has a Karnofsky performance score of 90. Current MRI with gadolinium is shown (A: axial; B: sagittal; C: coronal). Possible treatment choices were: surgical resection, chemotherapy and radiotherapy, best supportive care.

**Fig. 2:**
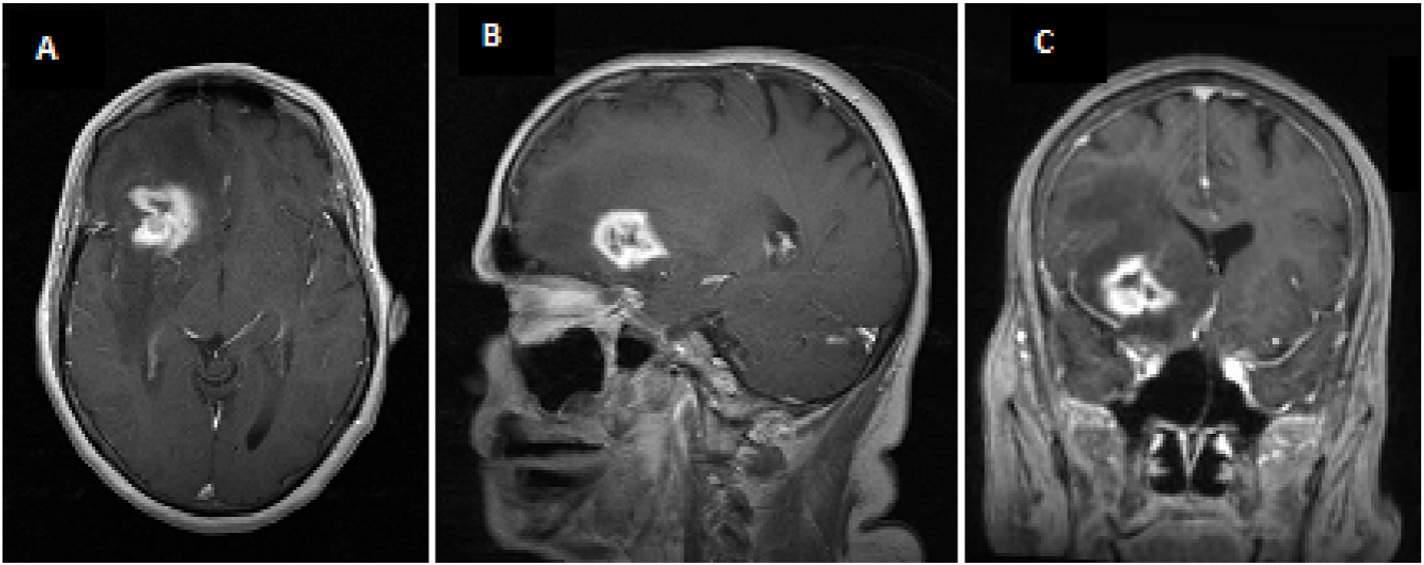
nMeta case - A 55-years old female presents with a recurrent solitary brain metastasis from a mamma carcinoma, six months after resection and local fractionated radiotherapy. Patient has otherwise stable disease without other metastases. She has a Karnofsky Performance Score of 90. Current MRI with gadolinium is shown (A: axial; B: sagittal; C: coronal). Possible treatment choices were: surgical repeated resection, systemic or immunotherapy, best supportive care.

**Fig. 3:**
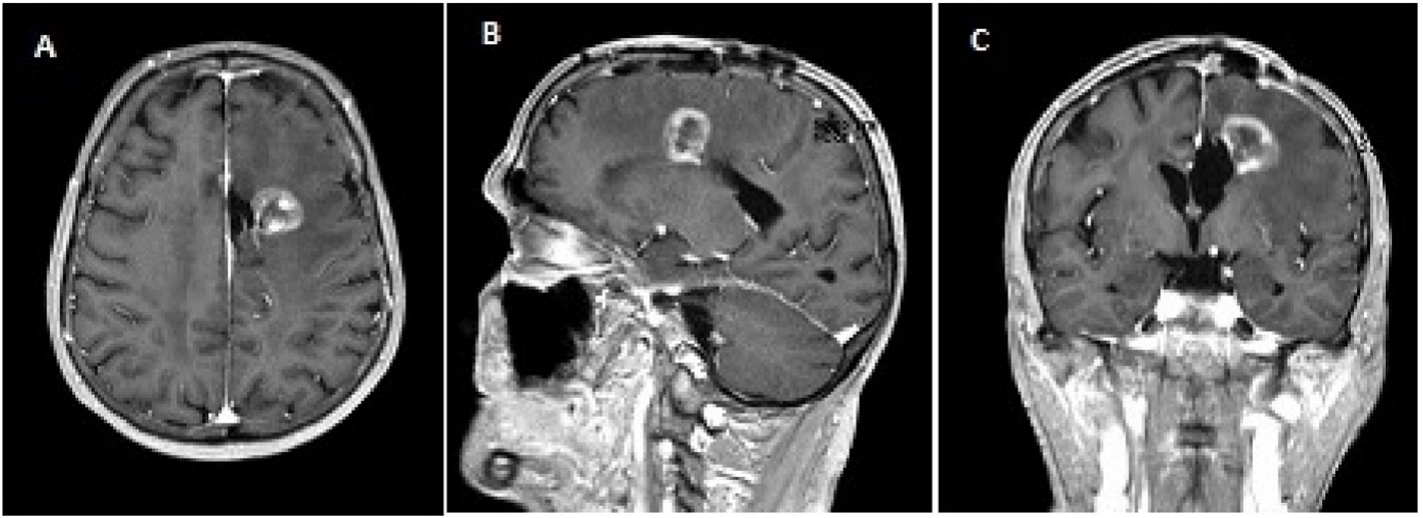
rGBM case - A 55-years old male presents with recurrent glioblastoma, six months after surgical resection and temozolomide/radiotherapy (Stupp protocol). No targetable mutations were found. Patient has a Karnofsky Performance Score of 90. Current MRI is shown. Possible treatment choices were: repeated resection, second line chemotherapy, best supportive care.

A survey comprising of 26-questions was mailed on 11 February 2019 using an online survey program (www.surveymonkey.com) to members of the European Association of Neurosurgical Societies (EANS), within the neuro-oncology section. Reminder emails were sent 3, 6 and 9 weeks later to initial non-responders to improve the response rate. The survey was divided into three sections. In the first section, we collected information regarding respondents’ demographics and general information. In the second section, we asked which indications were most suitable for SLA. Finally, we proposed three clinical cases: one patient with a newly diagnosed deep-seated glioblastoma (nGBM case; Fig. 1), one patient with a recurrent metastasis (rMeta case; Fig. 2) and one patient with a recurrent glioblastoma (rGBM case; Fig. 3). For all three cases we suggested three possible treatments most in line with the current European guidelines (Weller et al., 2014; Weller et al., 2020), asked participants to rate them according to their clinical practice and whether they would consider SLA for each case. We asked what effect they would expect using SLA with respect to overall survival (OS) and quality of life (QoL) in comparison to their current treatment of choice on a visual analogue scale from −100 to +100 (intending 0 as the same effect, +100 as positive effect, −100 as negative effect).

All data were analysed anonymously. Descriptive statistics were used as appropriate. Cross-tabulations with Chi-square analysis was used to identify association between respondents’ characteristics and their answers. SPSS version 25.0 was used to perform analyses.

## 3 Results

Of the 1446 neurosurgeons members of the EANS who were invited to participate, 128 respondents started the survey and 110 completed all the questions (9% response rate and 86% completion rate). Partially completed surveys (18) were included in the analysis up to the last completed question.

### 3.1 Demographics

The median years of experience in neuro-oncology of respondents was 10 years (range 1-45y). 40% (51/128) of respondents were working in an academical hospital, 24% (31/128) in a non-academical hospital, and 36% (46/128) had a combined appointment in both academic and non-academic hospital. The majority of respondents (58%, 74/128) worked for a centre where approximately 100–500 neuro-oncological patients are treated every year, 31% (40/128) for a centre with less 100 patients per year, and 11% (14/128) for a centre with more than 500 patients.

### 3.2 Indications for SLA

**Fig. 4:**
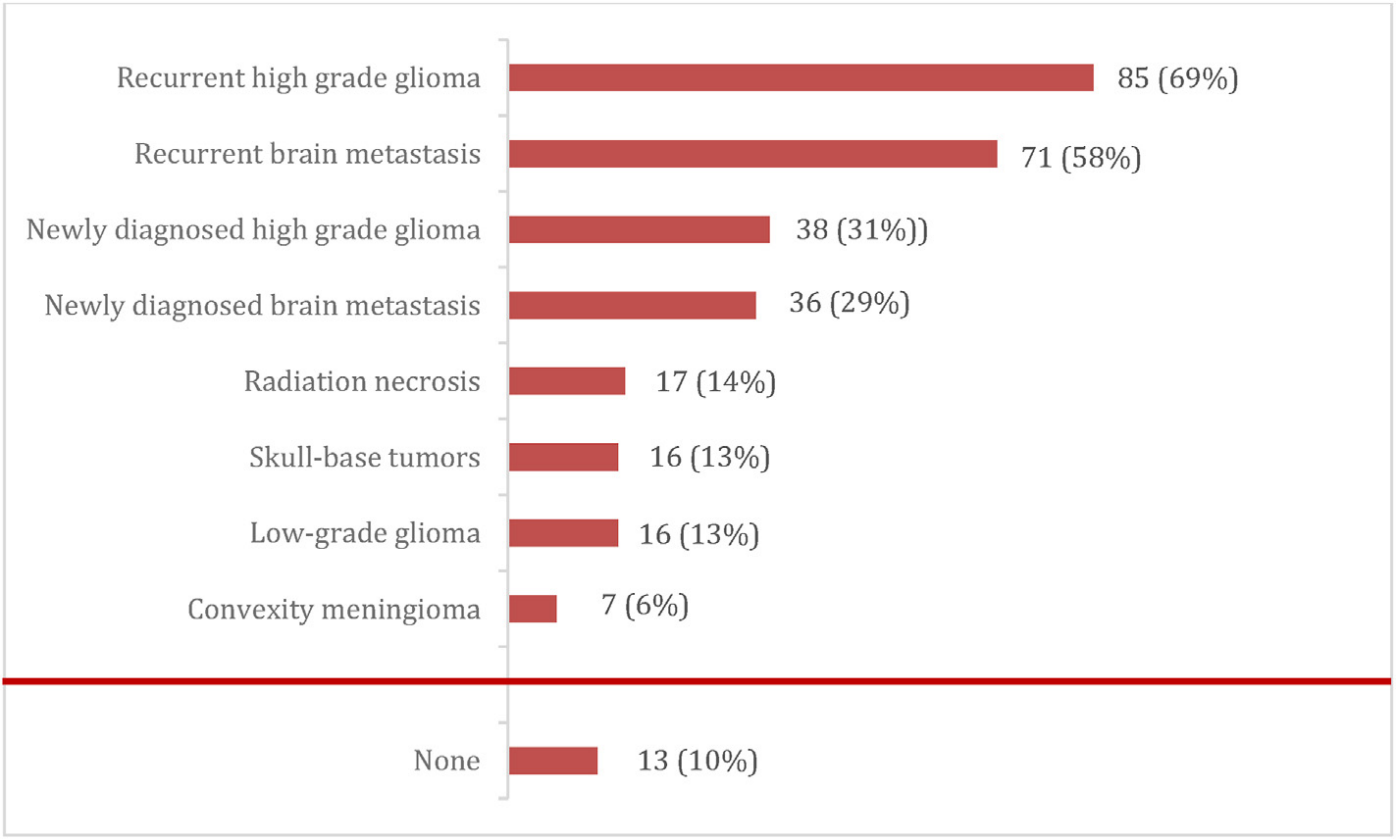
Overview of indications for SLA according to our respondents. Multiple answers were possible. Number of respondents: 123.

The majority (94/123, 76%) of the respondents had heard of SLA before the survey. Recurrent glioblastoma and recurrent metastases were regarded as the most feasible indications (chosen respectively by 85, 69%, and 71, 58%, respondents) followed by newly diagnosed high-grade gliomas (38 respondents, 31%) and newly diagnosed brain metastases (36 respondents, 29%) (Fig. 4). Thirteen respondents (13/123, 10%) found no valid indication for SLA.

Six respondents (6/123, 5%) reported to have SLA available as a treatment at their department. Of those who didn't, the majority (86/117, 74%) would refer patients for SLA. Of those who wouldn't refer patients for SLA most mentioned the current lack of evidence as reason for their reticence. Also, the costs of the procedure were mentioned as a limitation. We did not observe differences in respondents' answers according to their type of practice (academic, not academic, both), caseloads (<100, 100–500/>500) or years of experience (<5 years, 6–25 years, >25 years) (See Supplementary material).

### 3.3 Clinical cases

For the nGBM case, the majority of respondents (90/110, 82%) chose chemotherapy and radiotherapy as first treatment option. Fourteen percent (15/110) opted for surgical resection first, and 4% (5/110) chose best supportive care only.

For the rMeta case, the majority of respondents chose re-resection as first treatment option (87/110, 79%), 19% (21/110) systemic or immunotherapy, and 2% (2/110) best supportive care only.

For rGBM case, repeated surgery was the most chosen treatment (71/110, 65%), followed by second line chemotherapy (30/110, 27%). Best supportive care was chosen by 8% (9/110) of respondents.

**Fig. 5:**
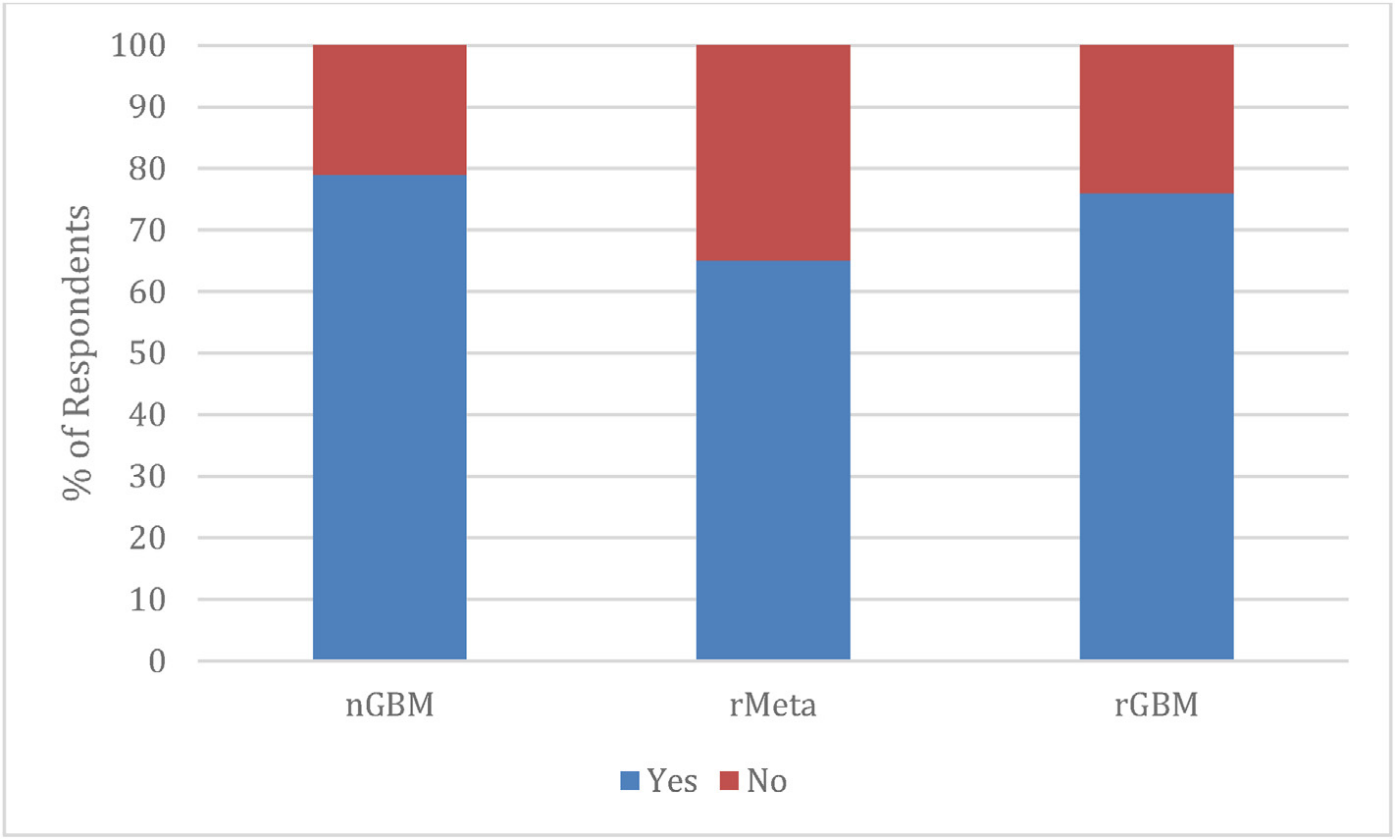
Would you consider SLA for the three cases? Number of respondents: 110. nGBM: newly diagnosed glioblastoma; rMeta: recurrent metastasis; rGBM: recurrent glioblastoma.

Seventy-nine percent (87/110) of respondents would consider SLA for the nGBM case, 65% (72/110) for the rMeta case and 76% (84/110) for rGBM case (Fig. 5).

Respondents who wouldn't consider SLA for the three cases (23/110) were asked to explain why in an open question. Overall, the most frequent reported reasons not to opt for SLA was the preference for standard therapy and the lack of evidence for SLA (respectively 56% and 26%), followed by risks of complication (9%) and other reasons (9%). We did not observe differences in respondents' answers according to their type of practice (academic, non-academic, both), caseloads (<100, 100–500, >500) or years of experience (<5 years, 6–25 years, >25 years) (see Supplementary material).

When asked about the potential effect of SLA on survival in comparison to standard therapy on a scale from −100 (negative effect) to +100 (positive effect), respondents on average expected a moderate positive effect of SLA for nGBM (mean ​+ ​13.3, standard deviation, SD, ±35.6), a minimal (mean ​+ ​5.2, SD ​± ​36) positive effect for rGBM and same effect (mean ​+ ​0.2, SD ​± ​41.9) for recurrent metastases. For all three cases a small to moderate positive effect on QoL was expected. Average expected effect on QoL was highest for nGBM (mean ​+ ​13, SD ​± ​35.5) and rGBM (mean ​+ ​12.9, SD ​± ​35.5), while a relatively lower effect on QoL was expected for recurrent metastasis (+4 ​± ​41.6).

## 4 Discussion

The aim of our international survey was to explore the current opinion about the use of SLA in neuro-oncology among European neurosurgeons. The 128 respondents were neurosurgeons with a median of 10 years of experience, mostly working in an academical hospital. Most of them were already aware of the existence of SLA for treatment of brain tumours.

According to our respondents, recurrent high-grade gliomas and recurrent metastases stand out as the most suitable indications for SLA, followed by newly diagnosed gliomas and metastases. We found some variation in the management of three exemplar cases, particularly in the recurrent glioblastoma case. Practice variation is a well-known phenomenon in neurosurgery and the lack of high-quality evidence and guidelines are often reported as explanatory for this finding (Cook et al., 2018; van Essen et al., 2019; van Opijnen et al., 2023). Even though in neuro-oncology guidelines are available (Stupp et al., 2014; Weller et al., 2020; Le Rhun et al., 2021), physicians have to deal with patients’ individual differences, intensive treatments and often poor prognosis, whereas, especially for recurrency and inoperable tumours, the decision-making is a matter of careful multidisciplinary deliberation, resulting in patient-tailored treatments.

Most of our respondents would consider SLA as a treatment option for all three presented clinical cases (79% for the case of a deep-seated glioblastoma, 65% for the case of a recurrent brain metastasis, 76% for the case of recurrent glioblastoma). Interestingly, when asked about general indications for SLA, one third of respondents would consider it for nGBM, while the great majority would consider SLA for the presented clinical case of nGBM. This probably indicate that variability in indication exists depending on tumour location and volume and possibility to perform surgery safely.

Among respondents who wouldn't consider SLA, the preference for standard treatments and lack of clinical evidence are reported as the biggest concerns. In general, respondents expect SLA to perform equally or slightly better to standard therapy for overall survival and better for quality of life, with the highest expected positive effect for nGBM.

Even if results with SLA are often reported as promising, we should keep in mind that published series are small and substantial (selection) bias might be likely, given the retrospective nature of most of the published series. To date, no randomized controlled clinical trials comparing the efficacy of SLA with standard of care have been published.

Three recent systematic reviews have attempted to summarize current evidence about the use of SLA for the three most common indications (Alattar et al., 2019; Montemurro et al., 2020; Viozzi et al., 2021). The most relevant findings of these three reviews are summarized in Table 1, which show that the reported survival outcomes varied enormously. As data from randomized trials are lacking, the reported survival in all case-series might be biased and unreliable. A minority of our respondents would consider SLA also for skull-base tumours, convexity meningioma and low-grade tumours, but it should be noted that only sporadic case reports have been published describing the application of SLA for other indications (Patel et al., 2015, Tovar-Spinoza and Choi, 2016; Millesi and Wolfsberger, 2017).

**Table 1:** Summary of the most relevant outcomes of the three most recent systematic reviews about SLA. N ​= ​number. OS ​= ​overall survival. PFS=Progression free survival. rMeta ​= ​recurrent metastases. rGBM ​= ​recurrent glioblastoma. nGBM ​= ​newly diagnosed glioblastoma.

Authors	Year	Investigated population	Included articles (n)	Patients (n)	OS(mo,range)	PFS(mo,range)	Complications (%)	Mortality(%)
Alattar et al.	2019	rMeta	13	142	5.8–19.8	NR	NR	NR
Montemurro et al.	2020	rGBM	17	203	6.1–14	2.8–10	6.4%	0
Viozzi et al.	2021	nGBM	11	114	4.1–32	2–31	33.7%	4%

It is possible that the high willingness to treat patients with SLA despite clinical evidence reflects the poor prognosis and lack of promising treatments that these indications share. One might consider SLA in these cases because of lack of better options. Nevertheless, the development of new technologies gives great responsibility to physicians, researchers and medical device corporations involved in the process. Well-designed controlled trials and transparent protocols to assess (cost-) effectiveness of this novel technique in comparison to standard therapy are in our opinion essential to allow a growing base of evidence to support the use of SLA in neuro-oncology and to protect the interests and safety of patients. Since May 2021 updated regulations have been applied by the European Commission to ensure safety of medical devices, including reinforcement of the rules on clinical evidence and strengthening of post-market surveillance requirements for manufacturers (Commission, 2020). It is therefore imperative that both manufacturers and clinicians collaborate in providing the required evidence to appropriately assess the added value of new devices and treatments. We therefore argue that patients treated with SLA should be included in experimental investigations and/or data will be shared to allow improved decision-making, both for treating physicians as well as patients.

The major strengths of our survey comprise the relatively large number of participating European neurosurgeons, which provide an overview of the current opinions among neurosurgeons with respect to a new technique for which interest and applications are growing, despite the lack of high level clinical evidence.

Some possible limitations should also be discussed. First, the response rate was only 9% despite several reminders. Response rate to surveys is known to be low among physicians and especially among surgeons, with the response rate to our survey being in line with those reported by others (Thoma et al., 2011). Although the demographic characteristics of our respondents suggest they should be representative also for the majority non-responders, a bias cannot be precluded which might influence the generalizability of our results. Second, we presented a relatively small amount of questions and cases, in order to limit the time burden for respondents and improve response rate, which means we were able to collect a limited amount of information about respondents and investigate only few aspects of the topic.

## 5 Conclusions

Our survey among European neurosurgeons confirms an interest in SLA, particularly for patients without other treatment options. Most of our respondents would consider SLA as a treatment option for recurrent glioblastoma, recurrent metastases and newly diagnosed deep seated glioblastoma. At the moment, the clinical evidence to support this trend is very poor. Given the interest of European neurosurgeons and the increasing use of SLA in patients groups with no or few other options, well-designed prospective phase II and III trials comparing SLA with standard care with respect to survival, quality of life and cost-effectiveness are warranted. We would suggest only treating patients with SLA in a controlled study, in order to protect the interests and safety of patients and allow a growing evidence base to support treatment decisions.

## Statements and declarations

All authors disclose any financial interests that are directly or indirectly related to the submitted work.

## Declaration of competing interest

The authors declare the following interests/personal relationships which may be considered as potential competing interests: We (IV, CO, ML) have previously been supported (in kind and financially) by Medtronic (r) for performing a laser ablation pilot trial (Grant ERP-2020-12244). Currently all authors are involved in a government funded RCT on laser ablations in glioblastoma, NCT05318612.
