# Supplementary material for: Stereotactic laser ablation in neuro-oncology - A survey among European neurosurgeons
Source: Brain Spine. 2023 Apr 28;3:101749. doi: 10.1016/j.bas.2023.101749 (PMC10293215; doi:10.1016/j.bas.2023.101749)
Supplement: Multimedia component 1 [file mmc1.docx]

**Supplementary material**

|  | **Willingness to refer for SLA** | | |
| --- | --- | --- | --- |
| **Respondents** | **No SLA** | **Yes SLA** | **p-value** |
| **Experience** |  |  |  |
| <5 years | 6 (17%) | 27 (83%) | 0.4 |
| 6-25 years | 17 (27%) | 43 (73%) |  |
| >30 years | 8 (32%) | 16 (68%) |  |
| Total | 31 (26%) | 86 (74%) |  |
| **Case-load** |  |  |  |
| <100 | 5 (14%) | 30 (86%) | 0.1 |
| 100-500 | 22 (31%) | 49 (69%) |  |
| >500 | 4 (36%) | 7 (64%) |  |
| Total | 31 (26%) | 86 (74%) |  |
| **Practice** |  |  |  |
| Academic | 13 (29%) | 32 (71%) | 0.6 |
| Both | 9 (21%) | 34 (79%) |  |
| Non-academic | 9 (31%) | 20 (69%) |  |
| Total | 31 (26%) | 86 (74%) |  |

Willingness to refer patients for SLA and respondents’ demographics, crosstabulation with Chi-square analysis. Total responder: 117.

|  | **Willingness to refer for SLA** | | | | | | | | |
| --- | --- | --- | --- | --- | --- | --- | --- | --- | --- |
| **Respondents** | **Case nGBM** | | | **Case rMeta** | | | **Case rGBM** | | |
| **Experience** | **No SLA** | **Yes SLA** | **p- value** | **No SLA** | **Yes SLA** | **p- value** | **No SLA** | **Yes SLA** | **p- value** |
| <5 years | 8 (24%) | 25 (76%) | 0.8 | 13 (40%) | 20 (60%) | 0.3 | 8 (24%) | 25 (76%) | 0.9 |
| 6-25 years | 10 (18.5%) | 44 (81.5%) |  | 15 (28%) | 39 (72%) |  | 12 (22%) | 42 (73%) |  |
| >30 years | 5 (22%) | 18 (78%) |  | 10 (43.5%) | 13 (56.5%) |  | 6 (26%) | 17 (74%) |  |
| **Case-load** | **No SLA** | **Yes SLA** | **p- value** | **No SLA** | **Yes SLA** | **p- value** | **No SLA** | **Yes SLA** | **p- value** |
| <100 | 4 (12.5%) | 28 (87.5%) | 0.1 | 8 (25%) | 24 (75%) | 0.4 | 7 (22%) | 25 (78%) | 0.7 |
| 100-500 | 14 (21%) | 52 (79%) |  | 25 (38%) | 41 (62%) |  | 15 (23%) | 51 (77%) |  |
| >500 | 5 (42%) | 7 (58%) |  | 5 (42%) | 7 (58%) |  | 4 (33%) | 8 (67%) |  |
| **Practice** | **No SLA** | **Yes SLA** | **p- value** | **No SLA** | **Yes SLA** | **p- value** | **No SLA** | **Yes SLA** | **p- value** |
| Academic | 10 (23%) | 33 (77%) | 0.9 | 19 (44%) | 24 (56%) | 0.2 | 8 (19%) | 35 (81%) | 0.2 |
| Both | 8 (19%) | 34 (81%) |  | 11 (26%) | 31 (74%) |  | 9 (21%) | 33 (79%) |  |
| Non-academic | 5 (20%) | 20 (80%) |  | 8 (32%) | 17 (68%) |  | 9 (36%) | 16 (64%) |  |

Willingness to refer for SLA and respondents’ demographics, crosstabulation with Chi-square analysis. Total responder: 110. rMeta= recurrent metastases. rGBM= recurrent glioblastoma. nGBM= newly diagnosed glioblastoma
